# Chemokines as therapeutic targets for multiple sclerosis: a spatial and chronological perspective

**DOI:** 10.3389/fimmu.2025.1547256

**Published:** 2025-03-21

**Authors:** Nagisa Nakata Arimitsu, Alicja Witkowska, Ayaka Ohashi, Chie Miyabe, Yoshishige Miyabe

**Affiliations:** ^1^ Department of Immunology and Parasitology, St. Marianna University of School of Medicine, Kawasaki, Japan; ^2^ Department of Sleep Medicine and Metabolic Disorders, Medical University of Lodz, Lodz, Poland; ^3^ Department of Frontier Medicine, Institute of Medical Science, St. Marianna University of School of Medicine, Kawasaki, Japan

**Keywords:** multiple sclerosis (MS), experimental autoimmune encephalomyelitis (EAE), chemokines, CNS (central nervous system), immune cells, therapeutic targets

## Abstract

Multiple sclerosis (MS) is a chronic autoinflammatory disease of unknown origin, involving characterized by immune cell infiltration into the target tissue, central nervous system (CNS), resulting in local and/or systemic inflammation. The symptoms vary from gait disturbance, visual impairment and learning and memory impairment and are being managed with corticosteroid and/or immunosuppressive agents. However, several patients do not respond to these treatments, which can also elevate the risk of severe infections. Therefore, there remains an ongoing need to identify new therapeutic targets. MS exhibits distinctive pathology, clinical course, and treatment responses, suggesting the importance of targeting disease site-specific immune cells to mitigate immune system-induced inflammation, rather than employing broad immunosuppression. Chemokines and chemokine receptors play a crucial role in the pathogenesis of MS by recruiting immune cells to the CNS, leading to inflammation and demyelination. Therapies targeting chemokines have shown promising results in preclinical studies and clinical trials, but more research is needed to fully understand their mechanisms and optimize their efficacy.

## 1 Introduction

Multiple sclerosis (MS) is a chronic autoinflammatory disease of unknown pathogenesis characterized by immune cell infiltration into target tissue, especially the central nervous systems (CNS), leading to local and/or systemic inflammation. This infiltration results in subsequent axonal damage, demyelinating inflammation, and the formation of sclerosing plaques in brain tissue (1, 2). In general, corticosteroid and/or immunosuppressive agents are among the first line treatment for MS patients with CNS involvement. Nevertheless, some patients fail to respond to these agents, and their usage carries an elevated risk of severe infection (3, 4). With the primary objective of therapy being to reduce the frequency of relapses, limit the accumulation of persistent disability, and prevent or delay the onset of progressive disability, there remains an urgent need to identify new treatments for MS, one potential avenue being the targeting of chemokines.

Chemokines constitute a family of small chemotactic cytokines crucial for regulating leukocyte migration during inflammation. They initiate intracellular signaling cascades that drives processes such as cell polarization and adhesion. “Classical” chemokine receptors, characterized by four conserved cysteine residues arranged in CXC, CC, CX_3_C, and XC patterns, are categorized into four subclasses based on their structural configuration. These receptors, expressed on the surface of immune cells, bind to chemokines and activate intracellular signaling pathways that promote cell polarization, adhesion, and migration. Classical chemokine receptors function as G protein-coupled transmembrane receptors (GPCRs). Upon chemokine binding, they initiate a series of intracellular signals that facilitate cell migration from the circulation into inflamed tissues or extravascular spaces (5–7). This migration process plays a pivotal role in various biological responses, particular in immune reactions, aiding immune cells in homing to sites of inflammation or infection.

Additionally, “atypical” chemokine receptors exist to regulate chemokine levels by removing them, thus suppressing inflammation. These receptors play a vital role in chemokine biology by capturing, scavenging, or transporting chemokines, thereby modulating their signaling through classical chemokine receptors (8).

Chemokines and chemokine receptors, pivotal in regulating leukocytes migration into tissue, play a crucial role in the pathogenesis of MS. Many studies have reported their involvement in recruiting of immune cells to the CNS, leading to CNS inflammation and demyelination. Thus, targeting these molecules has emerged as a promising therapeutic strategy for MS (7, 8). Further research is essential to fully understand the mechanisms behind these therapies and optimize their efficacy, given the complex relationship between immune regulation and MS pathophysiology. Therefore, this review provides a comprehensive overview of MS immunopathology, disease progression, and current treatment approaches drawing insights from analogous mechanisms observed in animal models. Additionally, we discuss the clinical effects of drugs with CNS involvement and their potential as new targets for MS with present up-to-date opinions in this regard.

## 2 Pathological findings in MS based on patients and mouse model

**Figure 1:**
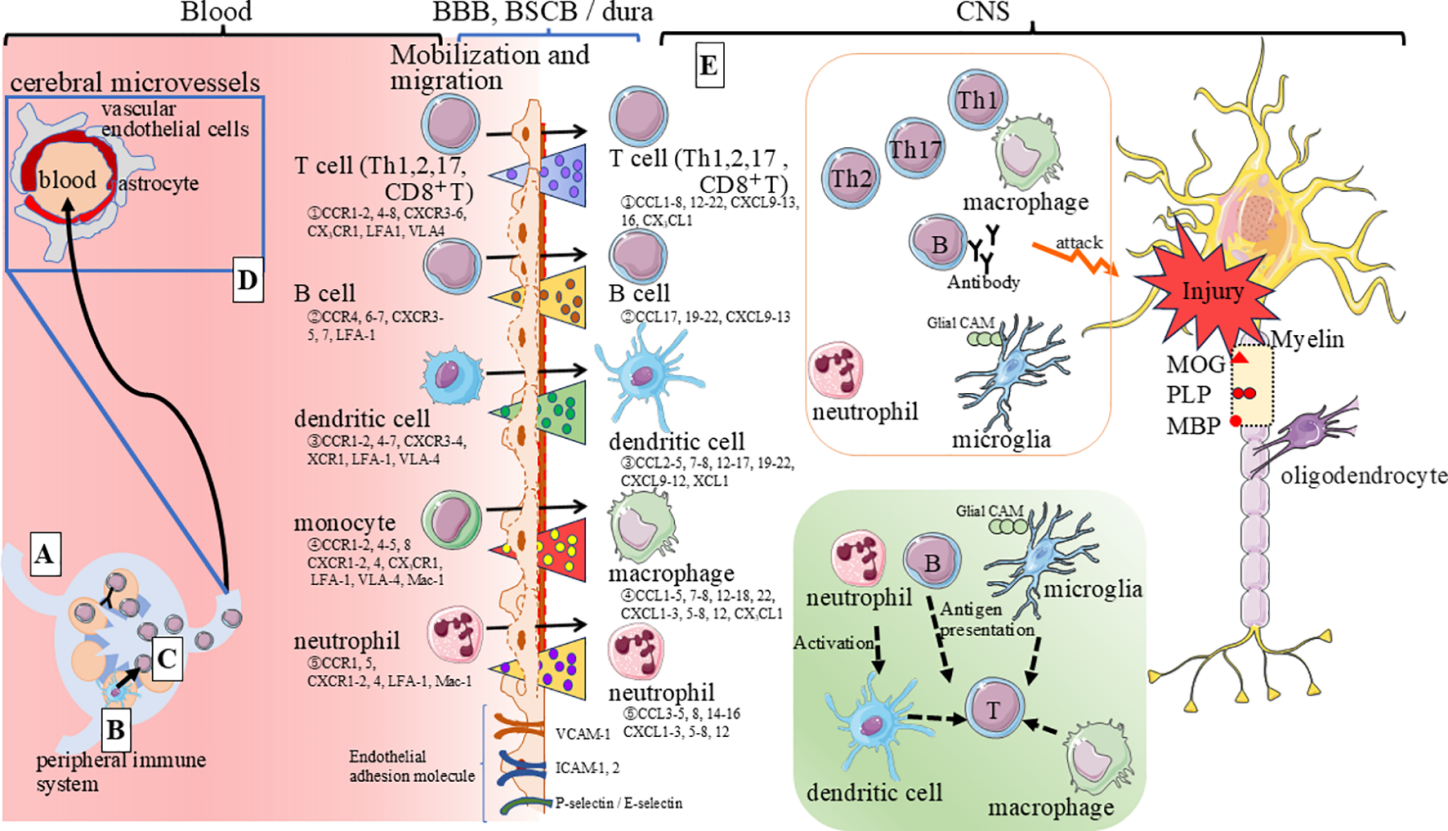
Estimated targets of disease-modifying therapies in MS. This figure illustrates the factors involved in immune cell migration and transition from peripheral tissues to the CNS in MS. The process begins with self-antigen activation, triggering immune responses. Helper T cells then migrate into the CNS, initiating pathological processes. This infiltration is regulated by interactions between immune and endothelial cells, mediated by chemokines and adhesion molecules. Autoantigen-specific T cells activate in the periphery, migrate to the CNS, and reactivate upon encountering APCs, initiating MS-like autoimmune processes. The figure depicts the following key steps: **(A)** Antigen Presentation in Lymph Nodes: Dendritic cells (DCs) and macrophages, activated by exposure to self-myelin antigens (e.g., MOG, MBP, PLP), migrate to lymph nodes as APCs. **(B)** Immune Cell Activation: Immune cells are further activated in peripheral lymph nodes as autoantigen-specific cells. **(C)** Migration to the CNS: Activated myelin-specific T cells (and sometimes B cells) enter the bloodstream and migrate toward the CNS. This process is facilitated by specific adhesion molecules such as intercellular adhesion molecule-1 (ICAM-1) and leukocyte function-associated antigen-1 (LFA-1). **(D)** Blood-Brain Barrier Disruption: Inflammatory cytokines produced by activated immune cells disrupt the BBB, BSCB, allowind immune cells to invade the CNS. This disruption is enhanced by expression of specific chemokines. Pro-inflammatory cytokines disrupt the BBB, enhancing leukocyte-endothelial adhesion. Th17 cells, known for their pro-inflammatory role, are increased in peripheral blood and CNS lesions. IL-17, produced by Th17 cells, disrupts BBB tight junctions, facilitating their transmigration across endothelial cells. Cytokines released by Th1 cells, such as IFN-γ and TNF-β, can activate macrophages, causing damage to oligodendrocytes and pathological changes in myelination. **(E)** Autoimmune Processes in the CNS: Upon encountering APCs presenting self-antigens in the CNS, these cells initiate MS-like autoimmune processes, such as axonal injury and myelin loss. This is accompanied by the release of chemokines that activate and mobilize other inflammatory immune cells.

The precise triggers for immunocompetent cell activation in patients with MS remain largely unknown. However, given that MS lesions are predominantly observed in the CNS, and HLA-DRB1*15:01 alleles are major susceptibility genes for MS (9), T cells and B cells have been implicated in the pathogenesis of MS (10). In patients with MS, CSF analysis often reveals mild pleocytosis with a clear predominance of lymphocytes over neutrophils (11). These immune cells are thought to react with various myelin antigens, including myelin basic protein (MBP), myelin oligodendrocyte glycoprotein (MOG), and proteolipid protein (PLP) (10). Recent research has shed light on the role of viral infections, particularly Epstein-Barr virus (EBV), in MS development. Longitudinal studies have shown a significant increase in MS risk following EBV infection, but not that with cytomegalovirus infection (12). Intriguingly, clinical studies have demonstrated symptomatic and objective improvement in MS patients following *in vitro*-expanded autologous EBV-specific T cell administrations (13). Notably, the efficacy of this therapy was associated with high EBV reactivity of the T cell product and downregulation of CSF IgG levels. Furthermore, molecular mimicry between EBV nuclear antigen (EBNA)-1 and CNS protein, a glial cell adhesion molecule (glialCAM) (**Figure 1**) was observed, leading to the production of a cross-reactive antibodies by pathogenic B cells (14). This finding suggests a promising clinical strategy for treating MS patients. Pathogenic roles of immune cells on MS are shown (**Figure 1**). During the early stages of inflammation, self-antigen activation triggers immune responses. Subsequently, helper T (Th) cells migrate into the CNS through the vasculature, initiating pathological processes (15–17) (**Figure 1**). The CNS has several barriers to prevent leukocyte infiltration. These barriers include the blood brain barrier (BBB) and blood-spinal cord barrier (BSCB), and the blood-cerebrospinal fluid barriers (BCSFB), which limit the entry of T cell entry into the CNS. The interaction between immune cells and endothelial cells, mediated by chemokines and adhesion molecules such as P/E selectins, Mac-1, LFA-1 (Lymphocyte function-associated antigen 1), VLA-4 (α4-β1 integrin), ICAM (Intercellular Adhesion Molecule) -1,2 and VCAM -1 (vascular cell adhesion molecule 1), is crucial for regulating leukocyte infiltration (18).

Autoantigen-specific T cells are initially activated in the periphery and subsequently migrate to the CNS, where they encounter antigen-presenting cells (APCs) presenting the autoantigen. This reactivation in the CNS initiates autoimmune processes similar to MS, accompanied by the release of chemokines that activate and recruit other inflammatory immune cells.

Post-mortem examinations have frequently revealed the accumulation of both T cells and B cells in the perivascular cuffs within the meninges and brain parenchyma of MS patients (16, 19).

In these tissues, the degree of cell infiltration are often associated with demyelination and neurodegeneration (19). Exposure of endothelial cells to proinflammatory cytokines such as IFN-gamma, TNF-alpha and IL-1beta, IL-17 etc. disrupts the BBB by disorganizing cell-cell junctions, reducing the brain solute barrier, and enhancing leukocyte-endothelial adhesion (20). Notably, Th17 cells, known for their proinflammatory role, are increased in both peripheral blood (16, 21, 22) and CNS lesions of MS patients, particularly those with active disease (17). IL-17 produced by Th17 cells disrupts BBB tight junctions, facilitating their transmigration across endothelial cells *in vitro* and *in vivo* (15).

Using animal models in research offers several advantages, including their physiological and pathobiological similarities to humans, well-characterized genomes and immune responses, and appropriate experimental size. Despite these advantages, challenges persist regarding reproducibility and translating findings to human disease models. One major difference is that EAE is induced by active immunization with CNS antigens, whereas the etiology of MS appears to be a complex interplay of genetic and environmental factors.

However, animal models remain valuable tools for investigating specific research questions. For instance, in studying MS pathogenesis, mouse models such as EAE induced in C57BL/6 or SJL/J mice using MOG or PLP peptides demonstrate molecular mimicry as a disease model for MS (23). The SJL/J strain specifically models relapsing–remitting MS. Additionally, genetic susceptibility or resistance to EAE in MHC congenic mice is associated with differential cytokine production. Antigens used in rodent EAE models include spinal cord homogenate (SCH), purified myelin, myelin proteins such as MBP, PLP, and MOG, or peptides derived from these proteins. The resulting disease phenotype varies from monophasic to relapsing-remitting or chronic EAE depending on the antigens and genetic background, and can be easily examined from the time of antigen stimulation (24). MHC background largely determines disease susceptibility, while epigenetic factors influence EAE incidence, onset time, severity, neurological signs, and CNS lesion distribution (25, 26).

## 3 Spatial and temporal MS treatment chemokine targets

The expression of several chemokines and their receptors is significantly elevated in the blood and inflamed tissues of MS patients, as well as in animal models of EAE. Considering the functions of chemokines, this suggests a pivotal role for chemokines in recruiting leukocytes and initiating inflammation in MS (27–31). The temporal dynamics of chemokine expression in EAE offer valuable insights into disease progression of the disease and the identification of potential therapeutic targets. Chemokines are produced by various cell types, but the specific cells involved vary depending on the context and location in the body. In blood, chemokines are primarily produced by immune cells, endothelial cells, and fibroblasts. In CSF, they are synthesized by ependymocytes, immune cells, and interstitial cells lining the ventricles. Within the CNS, chemokines are produced by cells forming brain blood vessels, as well as lymphocytes, macrophages, and microglia, particularly in active demyelinating MS brain lesions under pathological conditions. This process enables chemokines to induce and activate leukocyte adhesion molecules locally in certain tissues, establishing a chemotactic concentration gradient and contributing to endothelial permeability and monolayer recruitment (29, 30, 32, 33).

In the EAE animal model, the disease progresses through three distinct stages: the pre-symptomatic acute phase, the onset to disease phase (peak phase), and the chronic phase. Chemokine expression typically follows this pattern:

**Pre-symptomatic Acute Phase:** The disease manifests approximately 8 days after EAE induction in animals. Before onset, no visible motor impairments are observed, but an increase in T cells in the blood precedes the T cell infiltration into the CNS. Temporary vascular leakage in the cortical gray matter is observed within the first several days of disease induction, initiating microglial activation, followed by continuous accumulation of dendritic cells, leading to the infiltration of myelin-specific T cells (34–36). M-CSF1 (Macrophage Colony-Stimulating Factor) is upregulated in CSF, blood and CNS tissue as early as one day post-immunization (DPI). Before the peak onset of clinical symptoms, the expression of several chemokines, including CCL2, CCL3, CCL5, CXCL1, CXCL2, CXCL10, and CXCL11 increases in the CNS of EAE animals (37–41). For example, spatial variations in chemokine concentrations, driven by differences in expression in various CNS regions and in CSF versus serum (e.g., suppression of brainstem CXCL2 expression by IFN-γ and localized variations in CCL2 expression), have been shown to influence BBB integrity and recruit neutrophils and monocytes to the CNS (29, 33, 42). The IFN-γ pathway plays a significant role in chemokine localization from the spinal cord to the brain. EAE phenotype of IFN-γ KO mice exhibit altered patterns of chemokine expression in the spinal cord and brain, with a greater tendency for inflammatory cell infiltration (43).
**Onset Phase (Peak Phase):** The onset phase of EAE occurs approximately 8-15 days post-immunization, during which clinical symptoms reach their peak. During this period, CCL2, CCL3, CXCL1, and CXCL10 remain elevated. Additionally, proinflammatory cytokines, such as IL-1β, IFN-γ, CCL5, CCL12, and CXCL9 are upregulated in the spinal fluid, blood, and CNS of EAE animals (44, 45).
**Chronic Phase:** The chronic phase of EAE occurs approximately 15-30 days post-immunization. Following the peak phase, inflammation and demyelination begin to subside, leading to partial recovery in the animals, depending on the antigen type. During this phase the expression of cytokines such as M-CSF1, CCL2, CCL3, and CCL5 decreases (29, 38, 46). This reduction is associated with the resolution of inflammation and diminished mononuclear cell infiltration into the CNS. However, nerve damage still remains and chronic neurologic symptoms persist. The chronic phase may last the animal’s entire life and results in progressive neurologic dysfunction. Persistent inflammation in the CNS can lead to further recurrence and further neurological deficits (29).

Chemokine ligands and receptors are considered potential risk factors for MS, with their expression linked to clinical disease activity and severity. Inhibiting the chemokine system has been shown to suppress MS inflammation and potentially prevent the onset of MS (**Tables 1a**, **b**). Consequently, controlling immune cell recruitment to the CNS has become a major therapeutic target.

**Table 1a:** Chemokine targets for MS and phenotypes of chemokine ligand gene-modified mice in MS models.

Gene	KO Mouse Phenotypes in EAE (Antagonist Treatment in MS Model and Phenotypes)
CCL2	Anti-CCL2 neutralizing antibody reduced mononuclear cell infiltration into the CNS and clinical severity in a mouse model of EAE (51, 58, 59). CCL2 KO mice exhibited less severe EAE clinical manifestations. In patients, CCL2 is expressed by astrocytes and macrophages in acutely demyelinating lesions and active chronic lesions. CCL2 levels in the CNS are reduced during increased disease activity in MS (60).
CCL3	Deletion of the CCL3 gene in Tregs results in partial disease protection in the MS mouse model. Anti-CCL3 treatment inhibited MNC recruitment into the CNS. Tregs deficient in CCL3 production fail to prevent (51).
CCL4	Tregs deficient in CCL4 production fail to prevent EAE progression. CCL4 expression in the CNS has been reported in the EAE model, similar to clinical findings in MS (61).
CCL5	CCL5 KO mice have not been fully analyzed, but clinical indicators suggest involvement. CCL5 levels in CSF were higher in MS patients than in controls. CCL5 expression in the CNS has been reported in the EAE model, similar to clinical findings in MS. Modified CCL5 ligands are effective in controlling symptoms and neurodegenerative diseases (51, 62). Immunological neutralization of CCL5 in the EAE model has shown conflicting results (51, 63).
CCL7	CCL7 KO mice have not been analyzed, but clinical indicators suggest involvement (64, 65).
CCL8	CCL8 KO mice have not been analyzed, but clinical indicators suggest involvement (66, 67).
CCL11	CCL11 KO mice have not been analyzed, but clinical indicators suggest involvement (66, 67).
CCL13	CCL13 KO mice have not been analyzed, but clinical indicators suggest involvement (66). Elevated CCL13 levels in brain tissue and CSF can attract monocytes and stimulate lymphocytes to secrete inflammatory cytokines (68).
CCL17	CCL17 KO mice showed mildly reduced clinical scores (69, 70).
CCL18	CCL8 is a functional analog of human CCL18 in mice. Clinical indicators suggest involvement. One study suggested that CCL18 can inhibit chemotaxis via CCR1, CCR2, CCR4, and CCR5 (71)
CCL19	CCL19 and CCL21 KO mice (plt/plt mice) are resistant to EAE induction (72). Clinical indicators suggest involvement (66).
CCL20	Clinical phenotypes of EAE in the chronic phase were slightly exacerbated in CCL20 KO mice and may be compensated for by other chemokine signals (73). Clinical indicators suggest involvement (73, 74).
CCL21	CCL19 and CCL21 KO mice (plt/plt mice) are resistant to EAE induction (72). CCL21 and CXCR3 have functions in traumatic and EAE-induced neuropathic pain but are not involved in pathology (75).
CCL22	CCL22 KO mice have not been analyzed, but clinical indicators suggest involvement in MS pathogenesis in women (69, 76). Administration of anti-CCL22 at the time of autoantigen immunization delayed the initiation of clinical disease (77).
CXCL1	CXCL1 KO mice have not been analyzed, but clinical indicators suggest involvement (39). Anti-CXCL1 antibody reduced granulocyte adhesion to brain capillaries, and daily administration of this antibody for one week reduced EAE severity (78, 79)
CXCL2	CXCL2 KO mice have not been analyzed, but clinical indicators suggest involvement (33, 80).
CXCL5	CXCL5 KO mice have not been analyzed, but clinical indicators suggest involvement (39, 81)
CXCL8	CXCL8 KO mice have not been analyzed, but clinical indicators suggest involvement (79, 82–84).
CXCL9	CXCL9 KO mice have not been analyzed, but clinical indicators suggest involvement (33, 85)
CXCL10	CXCL10 KO mice developed severe MOG-induced EAE (86). The blocking CXCL10 function in rats increases EAE severity (87). However, clinical deficits were milder, and acute demyelination was substantially reduced in astroglia CXCL10-deleted EAE mice, but long-term axon loss was equally severe (88). The function of CXCL10 on EAE still remains unclear.
CXCL11	CXCL11 KO mice have not been analyzed, but clinical indicators suggest involvement (89, 90).
CXCL12	CXCL12 KO mice have not been analyzed, but clinical indicators suggest involvement. CXCR7 antagonist CCX771 prevented CXCR7, resulting in elevated abluminal levels of CXCL12, resulting in elevated abluminal levels of CXCL12 and reduced leukocyte infiltration, which ameliorated EAE severity (89, 91).
CXCL13	EAE onset occurs normally in CXCL13 KO mice, but disease severity wanes over time compared to wild-type mice (92, 93). Anti-human CXCL13 antibody, MAb5261, inhibited CXCL13-induced B cell migration.
CXCL16	CXCL16 KO mice have not been analyzed, but clinical indicators suggest involvement. Animals treated with anti-CXCL16 antibodies were resistant to EAE induction and showed decreased EAE severity (94, 95).
XCL1	XCL1 KO mice have not been analyzed, but clinical indicators suggest involvement. Animals treated with anti-XCL1 antibodies were resistant to EAE induction (96).
CX_3_CL1	CX_3_CL1 KO mice have not been analyzed, but clinical indicators suggest involvement (97). CX_3_CL1-mediated chemoattraction of NK cells is relatively specific for the CNS (98)

**Table 1b:** The phenotypes of chemokine receptors gene-modified mice in MS models.

Gene	KO Mouse Phenotypes in EAE (Antagonist Treatment in MS Model and Phenotypes)
CCR1	Deletion of the CCR1 gene results in partial disease protection in the MS mouse model. CCR1 antagonist ameliorates experimental autoimmune encephalomyelitis by inhibiting Th9/Th22-related markers in the brain and periphery (SJL/J) (99). In patients, CCR1 was detected on were detected on mononuclear cells and macrophages in demyelinating plaques (100).
CCR2	CCR2 KO mice did not illustrate CNS histopathology or clinical EAE (MOG induced in B6129PF2/J or C57BL/6J mice) and showed an obvious decrease in infiltrating T cells compared with control mice. CCR2 KO mice failed to develop EAE (59, 101). In patients, CCR2 was detected on infiltrating monocytes, macrophages and lymphocytes in MS lesions (54, 102).
CCR3	CCR3 KO mice have not been analyzed, but clinical indicators suggest involvement (103). In patients, CCR3 was detected on infiltrating monocytes, macrophages and lymphocytes in MS lesions (54, 102).
CCR4	CCR4 KO diminished clinical score of disease (104, 105). CCR4 and CCR6 double KO mice developed less severe EAE relative to control mice (69, 106).
CCR5	CCR5 KO mice did not protect against EAE. KO mice (B6129PF2/J genetic background) exhibited milder EAE with less severe T-cell infiltration and demyelination in the spinal cord compared with controls (51, 63, 107). A CCR5 receptor antagonist and antibodies to CCL20 have been reported to diminish disease severity (53, 54). In patients, CCR5 was detected on lymphocytic cells, macrophages, and microglia in actively demyelinating MS brain lesions and T cells expressing CXCR3 or CCR5 in CSF were increased (30).
CCR6	CCR6 KO mice or mice that were treated with a neutralizing anti-CCR6 antibody showed resistance to EAE development (108), CCR6 KO mice showed significantly more severe chronic EAE, but the pathological phenotypes of EAE in CCR6-KO mice were not consistent between the research groups (73, 106, 109). CCR4 and CCR6 double KO mice developed less severe EAE (69). In patients, T cells expressing CXCR3 and CCR6 in CSF were increased (30, 60).
CCR7	CCR7 KO mice acquired disease with an intensity similar to wild-type littermates. KO CD11c-eYFP cells infiltrated into the CNS but cells lacking CCR7 were retained in the brain and spinal cord while wild type DCs migrated to cervical lymph nodes (72, 110).
CCR8	CCR8 KO mice showed reduced EAE. CCR8 has been clearly demonstrated to play an essential role in EAE progression (111). CCR8 expression correlated with the demyelinating activity, but was not restricted to the MS pathology (112).
CXCR1	CXCR1 KO mice ameliorated disease severity in EAE mice (81).
CXCR2	Neutrophil-specific CXCR2 KO mice had less severe disease symptoms at peak and late phases when compared to control mice with similar levels of CNS-infiltrating neutrophils and other immune cells despite high levels of circulating CXCL1 (113).
CXCR3	CXCR3 KO mice developed severe MOG-induced EAE (114). In patients, T cells expressing CXCR3 in CSF were increased (30, 60) and CXCR3 was detected on infiltrating monocytes, macrophages, and lymphocytes in MS lesions (100).
CXCR4/CXCR7	CXCR4/CXCR7 KO mice have not been analyzed, but clinical indicators suggest involvement (89, 91, 115).
CXCR5	CXCR5 KO mice have not been analyzed, but clinical indicators suggest involvement (32, 93).
CXCR6	CXCR6 KO mice showed no protection, however, anti-CXCR6 mAb reverted EAE (32, 116, 117).
CX_3_CR1	While CX_3_CR1 KO mice showed severe MOG induced EAE and NK cells were obviously reduced in the inflamed CNS (98), the CX_3_CR1 inhibitor ameliorated EAE rat (118).
XCR1	XCR1 KO mice showed involvement in EAE development (96). XCR1 CAR-T cell-mediated depletion of DC1 modestly suppressed the onset of Th1-driven EAE (119).

Furthermore, due to functional overlap between chemokine systems, inhibiting a single chemokine may not be sufficient to completely suppress leukocyte migration. Therefore, targeting multiple chemokines and/or their receptors in combination may offer a more effective approach for treating human MS (5, 6).

## 4 Targeting the chemokine system in MS

The study of chemokines can 1) involve an influx of inflammatory cells from blood vessels, but since this occurs before the onset of symptoms, the timing and site-specific control of activity is possible from the outset, 2) provide potential lead compounds for MS therapy, 3) lead to a deeper understanding of MS pathogenesis, and 4) can be used therapeutically. The anti-inflammatory effect may be due to inhibition of chemotaxis, thereby preventing leukocyte influx into affected tissues. In addition, since there are multiple chemokines for each receptor, it is necessary to verify whether it is effective to inhibit them collectively or ligand-specifically. Chemokine activity can be controlled by inhibition against the chemokine itself or against the chemokine receptor, using peptide antagonists, neutralizing antibodies, and small non-peptide antagonists.

Immune neutralization of some chemokines [e.g., CCL3 acting on both CCR5 and CCR1, multiple chemokines sharing a single receptor, etc (47)] generally involves interactions with a variety of chemokine-receptors, so details of tissue localization and time-specific effects need to be identified. From both therapeutic and mechanistic perspectives, it is meaningful to administer selective small-molecule agents to block the effects of chemokine receptors on acute EAE and MS during the induction phase of the autoimmune response. **Table 1c** describes chemokines and receptors with potential therapeutic effects. We would like to discuss effective chemokine modulation for treatment by describing those that are affected by disease transition and protein/gene expression, those that are suspected to be involved in chemokines in gene-deficient animals, and those that are affected by chemokines in drugs already used in clinical practice.

**Table 1c:** The chemokine as the target for therapy of MS.

MS or EAE Targeting Agent/Drug	Target Cells	Modulated Chemokine/Chemokine Receptor	Supporting References
ACT-1004-1239	Various immune cells, oligodendrocyte precursor cells	CXCL12/CXCR7 (In the MOG-induced EAE model, ACT-1004-1239(10-100 mg/kg, twice daily, orally) showed a significant dose-dependent reduction in disease clinical scores and increased survival.)	(120)
Alemtuzumab	B cell, T cell on mature lymphocytes	CD52 (Approved for MS. Reduces accumulation of disability (≥1.0 point on EDSS) reduced by 66%, relapse rate by 72%, and by 55%. Reduces CCR3, CCR4, CCR5, CCR6, CXCR3, CXCL10, and CCL20.	(93, 94, 121, 122)
AMD3100	Various immune cells	CXCL12/CXCR4 (CXCL12 reduced in MS patient CSF cells and EAE spinal cord. Administration of antagonist AMD3100 to a weak EAE rats led to earlier disease onset. In mouse EAE, AMD3100 exacerbates disease by promoting leukocyte infiltration into the CNS parenchyma.)	(91, 123, 124)
Anti-CCL3 antibody	Macrophages, T cells	CCL3/CCR1, CCR5 (While CCL3 antibody treatment effectively suppressed EAE and prevented CNS mononuclear cell accumulation, Met-RANTES, a CCR1/CCR5 antagonist, only mildly decreased chronic-relapsing EAE severity without impacting leukocyte migration.)	(125, 126)
Anti-CXCL10 antibody	T cells, Eosinophils, Monocytes, Natural killer cells	CXCL10/CXCR3 (Paradoxically, while CXCL10 neutralization exacerbates EAE by increasing CD4+ cell infiltration in the CNS, it reduces inflammatory cell invasion, demyelination, and improves neurological function in a viral model of multiple sclerosis.)	(87, 127, 128)
Anti-CXCL16 antibody	T cells, NK cells, Dendritic cells	CXCL16 (Anti-CXCL16 antibody treatment confers resistance to EAE induction.)	(94)
Anti-CCR6 antibody	Th17 cells	CCR6 (Anti-hCCR6 treatment effectively reduced clinical EAE symptoms and decreased inflammatory cell infiltration within the central nervous system.)	(129, 130)
BTX/Evobrutinib	B Cells, Myeloid Cells	CCR1 (BTK inhibitor limits microglia-mediated inflammation *in vitro* and in multiple animal models of MS)	Phase III (131)
CCX771	Monocytes Oligodendrocytes	CXCL12/CXCR7/ACKR3 (CCX771 treatment ameliorates the clinical severity of EAE.)	(132, 133)
Cladribine	APCs, Peripheral B cells, Monocytes	CCL5, CXCL8. (Approved for relapsing MS. Decreases CXCL8 in CSF and CCL5 in CSF and serum. Blocks B and T cells, causing cell death.)	(134, 135)
Fingolimod	T cell, CD8^+^T cell	a sphingosine-1-phosphate receptor (S1PR) (Approved for MS. Sequesters lymphocytes in lymph nodes. Reduces annualized relapse rate reduced by 39% (1.25 mg) and 52% (0.5 mg). Reduces CXCR4-mediated B cell migration.)	(136–138)
Glatiramer acetate (GA)	Antigen-presenting cells (APCs)	CCR5, CCR7, CXCR3, CXCR6 (Approved for MS. Mixture of random-sized peptides that mimic myelin basic protein, acting as a decoy. Reduced relapse rate by 29%. Modulates CCR1, CCR2, CCR3, CCR7, CCR9, CCR10, CXCR1, CXCR4, CXCR5, CXCR6, and CCR6.)	(139)
Interferon-Beta (IFN-β)	B cells, Neutrophils	CCL2, CCL3, CCL5, CCL7, CXCL8, CXCL9, CXCL10/CCR5, CXCR3 (Approved for MS. IFN-β therapy suppresses MS disease activity. Decreases circulating neutrophils in RRMS patients, and showed a decrease in neutrophil infiltration in animal models. Reduced relapse rate reduced (7% for 1.6 MIU, 33% for 8 MIU). Increases relapse-free patients (23% for 1.6 MIU, 50% for 8 MIU). Decreases of CCR5 and CXCR3 expression on T cells. Induces transient CXCL10 increase. High-dose IFN-β suppress CCL17 in peripheral blood.)	(140, 141)
Mab 526I	B cells	CXCL13/CXCR5 (Mab 526I attenuates symptoms in experimental autoimmune encephalomyelitis.)	(142)
Methylprednisolone (MP)	Various immune cells	CXCL10, CXCL13, CXCR1, CXCR2, CXCR3 (The high concentration of CXCL10 in MS serum of patients was diminished to the initial value after therapy with intravenous methylprednisolone.)	(143)
Met-RANTES	T cells (especially Th1 and Th17 cells), Monocytes, Eosinophils	CCL5/CCR1, CCR5 (While active treatment showed no effect on acute-monophasic EAE, regardless of administration timing, Met-RANTES modestly improved fixed neurological disability when administered at disease onset in chronic-relapsing EAE.)	(126)
Mitoxantrone	T cells (autoreactive), B cells, Macrophages, APCs	CCL2, CCR2, CXCR1, CXCR2 (Intrathecal methotrexate slowed disability progression, decreased CXCR1 and CXCR2 expression on peripheral blood mononuclear cells.)	(144)
Natalizumab (Tysabri)	Leukocytes (α4-integrin expressing)	CCL22, CXCL9, CXCL10, CXCL11, CXCL13 (Approved for relapsing MS. α4 integrin antagonist, preventing leukocyte trafficking into the CNS. Reduces CSF levels of Th1 (CXCL9, CXCL10, CXCL11) and Th2 (CCL22) chemokines. Increases CXCR3-expressing B cells.)	(145)
NSC-87877	T cells	CXCL12/CXCR7 (NSC-87877 abrogated EAE by blocking initial CD8^+^ T-cell into the uninflamed CNS.	(146)
Rituximab (Ofatumumab) Chimeric anti-CD20 antibody	B cells	CCL19, CXCL8, CXCL10, CXCL13 (Approved for MS. Annualized relapse rate: 0.11 with ofatumumab, 0.22 with teriflunomide Modulates CXCL10.)	(147)

• CCR1 Antagonists

CCX354 is an orally administered, highly potent, and selective antagonist of the CCR1 receptor. In clinical studies, it has shown good tolerance and exhibited a linear dose-exposure relationship, with a half-life of approximately 7 hours at a 300-mg dose. Effective blockade of inflammatory cell infiltration into tissues requires maintaining ≥90% CCR1 inhibition on blood leukocytes at all times (48, 49). CCX354 was evaluated in two clinical trials for rheumatoid arthritis, where it demonstrated a favorable safety and tolerability profile, as well as clinical activity. The response rate at week 12 was 39% in the placebo group and 56% in the 200 mg once-daily group (p=0.01). To date, there is a lack of specific data on the use of CCX354 in MS. However, other CCR1 antagonists, such as CCX721, avacopan (CCX168), BMS-817399, and ASK-8007, show promise in addressing MS-related neuroinflammation (50). Notably, elevated CCR1 levels have been observed in cerebrospinal fluid (CSF) during the early and acute stages of demyelination episodes in MS. In an animal model of EAE, CCR1 expression was significantly reduced during clinical remission, correlating with decreased spinal cord inflammation and demyelination (51). This evidence supports the exploration of CCR1 antagonists as a potential therapeutic approach for MS.

• CCR2 Antagonists

CCX872, a CCR2 antagonist, targets the G-protein-coupled receptor CCR2, which is expressed on monocytes and macrophages and plays a crucial role in their migration and infiltration into tissues. Its anti-inflammatory potential was demonstrated using CX_3_CR1GFP/+CCR2RFP/+ reporter mice, where treatment with CCX872 effectively reduced the accumulation of peripheral macrophages, inhibited the neurotoxic polarization of these cells, and suppressed the expression of NOX2, a superoxide-generating enzyme that produces reactive oxygen species (ROS), within a day post-injury. Importantly, this intervention prevented traumatic brain injury (TBI)-induced deficits in hippocampal-dependent learning and memory observed 28 days after injury (52). Given the evidence that CCR2 is expressed on microglia and macrophages in chronic active MS lesions and on perivascular mononuclear cells in both white matter lesions and unaffected cortex, CCX872 presents a particularly promising candidate for further investigation in MS. However, if therapeutic intervention is considered, multiple targets must be addressed. This is because EAE in CCR2 knockout (KO) mice led to the replacement of neutrophils by monocytes, ultimately resulting in demyelination (53).

Dual Antagonists

• CCR2/CCR5

BMS-813160 is a dual antagonist of CCR2/CCR5 activation presenting with an excellent human liver microsome stability, oral bioavailability and low clearance in mouse, dog and monkey. It may inhibit inflammatory processes, angiogenesis, tumor cell migration, tumor cell proliferation and invasion and was clinically tested in this regard. Simpson et al.'s study, chronic active MS lesions were found to be associated with CCR2, CCR3, and CCR5, particularly in foamy macrophages and activated microglia, with CCR2 and CCR5 also being prevalent in infiltrating lymphocytes (54, 55). Given these findings, BMS-813160 might be a promising candidate for evaluation in MS treatment.

• CCR1/3 and CCR2/5

UCB 35625 and its enantiomer J113863 are known for their strong affinity towards CCR1 and CCR3, making them promising candidates for modulating immune responses. In preclinical studies using EAE model, daily administration of J113863 at a dose of 10 mg/kg for 12 days resulted in a significant reduction of pro-inflammatory CD4+GM-CSF+ and CD4+IL-6+ cells, while simultaneously increasing the levels of anti-inflammatory CD4+IL-27+ and CD4+IL-10+ cells in the spleen. Moreover, J113863 treatment was shown to suppress the mRNA and protein expression of pro-inflammatory cytokines GM-CSF and IL-6 in brain tissues, while enhancing the expression of anti-inflammatory cytokines IL-10 and IL-27. These findings suggest that CCR1 antagonists could play a valuable role in reducing neuroinflammation in MS. Interestingly, while UCB 35625 and J113863 primarily target CCR1 and CCR3, they also demonstrate low-affinity interactions with CCR2 and CCR5. Depending on the specific receptor, the enantiomer, and the signaling pathway involved, these compounds may act as antagonists, partial agonists, or full agonists. This versatility in function underscores the potential utility of these compounds in addressing the complex immune dysregulation observed in MS (56, 57).

## 5 Conclusions

This review synthesizes research on the role of chemokines in inflammatory diseases, with a particular focus on MS, and outlines recommended therapies and promising targets based on the disease mechanisms presented in **Figure 1**. Several chemokines and their receptors are implicated in MS pathophysiology, with by some being directly targeted or indirectly modulated by immunomodulatory agents and drugs (**Table 1c**). The distinctive pathology, clinical course, and treatment responses of MS underscore the importance of targeting disease site-specific immune cells to mitigate immune system-induced inflammation, rather than relying on broad immunosuppression. The pleiotropic nature of the chemokine system presents challenges for effective targeting with selective antibodies or receptor blockers. Furthermore, general chemokine blockade carries the risk of compromising host defenses, and discrepancies between findings in EAE models and species-specific differences in chemokine responsiveness necessitate careful consideration for clinical translation to human MS patients. These insights are valuable for developing novel therapeutic approaches in inflammatory diseases, especially in inhibiting the mobilization of pathogenic lymphoid cells within lesions and contribute to advancing therapeutic strategies for MS.
